# Acute Hepatitis A-Induced Autoimmune Hepatitis: A Case Report and Literature Review

**DOI:** 10.3390/medicina58070845

**Published:** 2022-06-24

**Authors:** Hye In Jo, Minchang Kim, Jeong-Ju Yoo, Sang Gyune Kim, Young Seok Kim, Susie Chin

**Affiliations:** 1 Division of Gastroenterology and Hepatology, Department of Internal Medicine, Soonchunhyang University Bucheon Hospital, Bucheon 14158, Korea; hyein525@naver.com (H.I.J.); mcnulty@schmc.ac.kr (S.G.K.); liverkys@schmc.ac.kr (Y.S.K.); 2 College of Medicine, Soonchunhyang University, Cheonan 31538, Korea; mckim970610@gmail.com; 3 Department of Pathology, Soonchunhyang University Bucheon Hospital, Soonchunhyang University School of Medicine, Bucheon 14158, Korea; susiechin@schmc.ac.kr

**Keywords:** acute hepatitis A, autoimmune hepatitis, jaundice

## Abstract

Introduction: The pathogenesis of autoimmune hepatitis (AIH) is little known. Previous case reports suggest that several viral hepatitis, including hepatitis A, can trigger AIH. Patient: A 55-year-old female showed general weakness and jaundice. The patient was diagnosed with acute hepatitis A and discharged after 14 days of hospitalization with improving liver function. However, blood tests performed 6 days after discharge revealed an increase in liver enzymes and high serum titers of an anti-nuclear antibody and immunoglobulin G. She was readmitted for liver biopsy. Diagnosis: Liver biopsy showed acute hepatitis A along with AIH. According to the revised international autoimmune hepatitis group scoring system, her score was 14 and she was diagnosed as AIH induced by acute hepatitis A. Intervention: Conservative treatments with crystalloid (Lactated Ringer’s Solution), ursodeoxycholic acid, and silymarin were administered. Outcomes: The patient has been followed up on an outpatient basis and neither symptom recurrence nor an increase in liver enzymes has been reported thus far. Lessons: After the treatment of acute hepatitis A, liver function needs to be carefully monitored over time, and the possibility of autoimmune hepatitis should be considered when liver enzymes increases.

## 1. Introduction

Autoimmune hepatitis (AIH) is a rare disease that causes chronic inflammatory changes in the liver, and the etiology of AIH is unknown [1]. It is characterized clinically by hypergammaglobulinemia and high serum titers of an anti-nuclear antibody (ANA), and histologically by portal inflammatory cell infiltration and piecemeal necrosis. Asialoglycoprotein-receptor-specific defects in T-suppressor-induced cells or human lymphocyte antigen (HLA) types are known as genetic contributing factors to AIH. However, its underlying mechanisms and triggers have not yet been clarified [2,3]. The pathophysiology of AIH has been continuously studied, and inflammatory cytokines have recently been identified as important mechanisms. For example, interleukin (IL)-17 is known to induce immune cell infiltration and liver damage, leading to hepatic inflammation and fibrosis and contributing to autoimmune liver diseases [4].

Some AIH case reports indicate that viruses causing acute hepatitis, such as the hepatitis A virus (HAV), hepatitis B virus (HBV), and Epstein–Barr virus, may trigger AIH [5]. Acute HAV infection is a common cause of viral hepatitis in humans and is usually self-limiting and resolves within a few weeks. The cause of acute HAV infection is the transmission of HAV, such as through the ingestion of contaminated food or water or direct contact with an infectious person. Although there is no clear evidence that HAV causes chronic liver disease, a few cases of an AIH occurrence after HAV infection have been reported. 

The current case report concerns a 55-year-old female patient with AIH that appeared to be triggered by acute HAV infection during the convalescent phase of acute hepatitis type A. Serologic and histologic findings were analyzed based on the clinical course, and the potential relevance of acute hepatitis type A to AIH is discussed.

## 2. Case Presentation

A 55-year-old woman who showed general weakness and jaundice visited a local clinic. She was referred from the clinic to an emergency room of a tertiary hospital with increased liver enzyme levels.

The patient had no medical or family history of hepatic disease. She regularly consumed 0.7 bottles (1 bottle: 360 mL) of soju once a week (alcohol 50 g/week). She was in her usual state of health until five days before the first visit to the local clinic, when she developed the acute onset of general weakness and jaundice. Acute hepatitis was presumed considering jaundice and increased liver enzymes. Her admission height was 158 cm, weight was 44 kg, and body mass index (BMI) was 17.62 kg/m^2^. Her blood pressure was normal, with a reading of 114/67 mmHg. Additionally, she had a normal body temperature (36.8 °C), heart rate (67 bpm), and breathing rate (18 breaths/min). 

Her laboratory findings were as follows: Aspartate aminotransferase (AST) of 3864 U/L, Alanine aminotransferase (ALT) of 4825 U/L, alkaline phosphatase (ALP) of 250 U/L, gamma-GTP (γ-GT) of 160 U/L, and total bilirubin of 4.08 mg/dL. Prothrombin time-international normalized ratio (PT/INR) was 1.92.

The results of blood tests related to hepatitis viral infection were as follows: Anti-hepatitis A virus IgM (anti-HAV IgM) (positive), hepatitis B surface antigen (HBsAg) (negative), anti-hepatitis B surface (anti-HBs) (positive), anti-hepatitis B core IgM (anti-HBc IgM) (negative), anti-hepatitis C virus (anti-HCV) (negative). A contrast-enhanced abdominal computed tomography (CT) scan showed heterogeneous parenchymal enhancement with diffuse periportal edema of the liver and subserosal edema of the gallbladder, suggesting acute liver disease. There was no obstructive lesion in the biliary tract.

**Figure 1:**
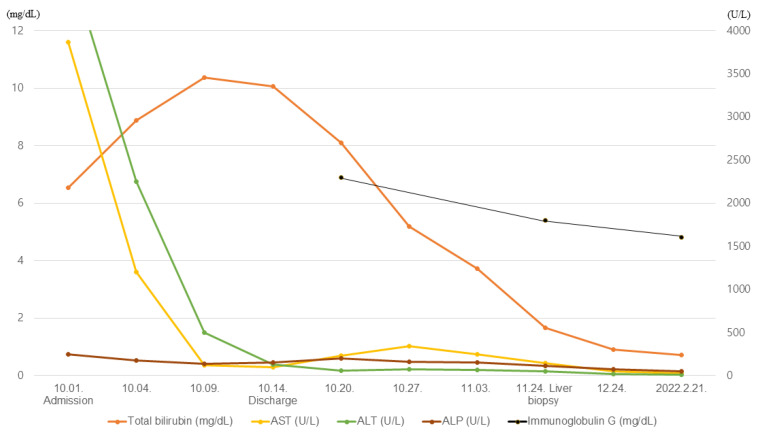
The clinical course of the patient.

After the conservative treatments of crystalloid (Lactated Ringer’s Solution), ursodeoxycholic acid, and silymarin, the patient was discharged on the 14th day of hospitalization with AST of 98 U/L, ALT of 129 U/L, total bilirubin of 10.07 mg/dL, and normal PT/INR (0.89). No medications other than those described above were administered during the hospitalization period (Figure 1). 

After discharge, the patient made three outpatient visits on the 6th, 13th, and 20th day, and her serum AST and ALT levels gradually increased as follows: AST/ALT of 234/56 U/L (6th day), 346/75 U/L (13th day), and 244/70 U/L (20th day). She tested positive for antinuclear antibody (1:160), while she was negative for anti-smooth muscle antibody (SMA), anti-liver kidney microsomal antibody (LKM), and anti-mitochondrial antibody (AMA). Liver biopsy showed acute hepatitis A along with AIH. 

**Figure 2:**
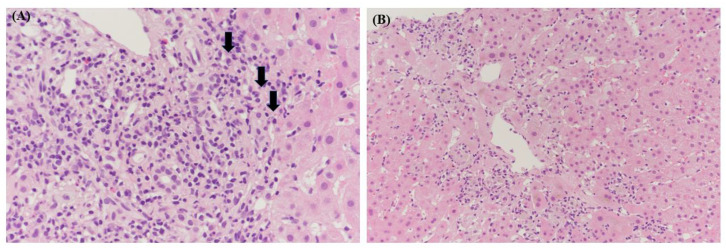
Pathologic findings of the liver biopsy specimen. (**A**) Histology showed lymphoplasmacytic infiltration in the portal tract with interface activity. Plasma cells are indicated with arrows. (Hematoxylin and eosin staining (H&E) × 200). (**B**) There is hepatocytic necrosis with cholestasis in the centrilobular area. (H&E × 100).

A clinical possibility was suggested that AIH was induced by hepatitis A based on the revised international autoimmune hepatitis group scoring system. The patient’s scoring details were as follows: being female (+2) and ALP: AST ratio of 0.85 (+2). Antinuclear antibody was 1:160 (+3) and immunoglobulin G (IgG) level was 2165 (+1). AMA was negative (0) and anti-HAV IgM was positive (−3). She had not used a hepatotoxic drug recently (+1) and drank alcohol less than 25 g per day (+2). Liver biopsy showed interface hepatitis (+3) and predominantly lymphoplasmacytic infiltration (+1). There was no rosetting of the liver cells and biliary change on liver biopsy (0). The final diagnosis based on liver biopsy was AIH induced by acute viral hepatitis A (Figure 2). After conservative treatment, her liver enzymes and IgG levels were normalized five months after the treatments, and she is currently under follow-up in outpatients (+2) (Figure 1). 

The study protocol was approved by the Institutional Review Board of Soonchunhyang University Bucheon Hospital (SCHBC 2022-05-015) and conformed to the ethical guidelines of the World Medical Association Declaration of Helsinki. The requirement for informed consent from the individual subject was waived due to the retrospective nature of the study.

## 3. Discussion

Liver enzyme alteration can be found in various liver diseases, but if you look closely, the common causes of liver disease have typical patterns [6,7]. For example, ischemic liver injury shows marked aminotransferase level (>1000 IU/L), and viral hepatitis or toxic hepatitis show slightly lower aminotransferase levels than the above disease. On the other hand, autoimmune hepatitis presents with a mild increase in aminotransferase levels with jaundice.

On average, 1–2 autoimmune hepatitis (AIH) cases are found for every 100,000 people per year in Norway and Sweden. Among AIH patients, 40% may experience acute onset of illness, and a severe acute presentation characterized by hepatic encephalopathy can occasionally take place within eight weeks after the clinical symptoms [8]. The underlying mechanism and pathogenesis of AIH are not yet elucidated. That being said, it is known that the cell-mediated immune system and the antibody-dependent mechanism that can damage liver are both attributed to autoantigen exposure, genetic predisposition, and immunoregulatory mechanism defect [9]. The genetic predisposition of type 1 AIH is associated with major histocompatibility complex (MHC) class II molecules HLA DR3 and DR4. In particular, patients with DRB1*0301 alleles are more prone to a more serious form of disease than patients who possess 0401 alleles [10,11]. The DRB1*0401 gene was identified from a case report of AIH after hepatitis A by Hilzenrat et al. [12] in 1999. The patient did not show progression to a severe state, which suggested that acute hepatitis A can trigger AIH in genetically susceptible patients. However, Tanaka et al. [5] compared HLA types in five case reports of AIH induced by hepatitis A, and no particular genetic tendency was identified. The prospective study by Vento et al. [13] indicated a possibility of the involvement of immunological abnormality in AIH after hepatitis A. In the study, they followed up 58 biological relatives of AIH patients for four years. Three of them caught hepatitis A, and two of the three developed AIH type1 within five months. Those patients had a defect in the suppressor T lymphocytes that regulate the immune response to the liver-specific autoantibody asialoglycoprotein receptor (ASGPR). Therefore, it was suggested that hepatitis A might be a trigger of AIH in immunosensitive patients.

According to the International Autoimmune Hepatitis Group (IAIHG), AIH is a type of chronic hepatitis characterized in liver biopsy by the serum autoantibody, hypergammaglobulinemia, interface hepatitis, and periportal plasma cell infiltration [14].

There are two types of AIH: types 1 and 2. Type 1 is common in adults, while type 2 is common in children and young women. Type 1 is characterized by serum antinuclear antibodies (ANA) or anti-smooth muscle antibodies (SMA), while type 2 is characterized by serum antibody to liver/kidney microsome (Anti-LKM) or antibody to liver cytosol-1 (Anti-LC1) [15]. In AIH diagnosis, the sensitivity and specificity of ANA are 65.0% and 75.1%, respectively, and the sensitivity and specificity of SMA are 59.3% and 92.6%, respectively [16].

It is difficult to differentiate AIH from other liver diseases because of the lack of characteristic symptoms and specific diagnostic methods. Moreover, autoantibodies in AIH are also found in other liver diseases. Therefore, in 1999, the original IAIHG scoring system was revised and it evaluates 15 parameters enabling the categorized diagnosis of AIH, from probable to definite [14].

For the treatment of AIH, caution needs to be exercised when the serum aminotransferase levels are more than five times the normal upper limits. Combination therapy of prednisone and azathioprine or monotherapy of high dose prednisone can be used. That being said, the combination therapy is preferred as an initial treatment option because of the low frequency of side effects [17]. Overall, 80–90% of patients with moderate/severe AIH showed decreased serum aminotransferase levels and improvement in symptoms within two weeks after the treatment. After remission, AIH recurrence (within 12 months) was found in 50–90% of the patients who were withdrawn from their drugs [18].

It has been reported that hepatitis A, B, C, D, and E viruses, cytomegalovirus, Epstein–Barr virus, measles virus, varicella-zoster virus, and human immunodeficiency viruses are associated with AIH [19,20,21,22,23,24,25,26,27,28,29]. In particular, the hepatitis A virus and Epstein–Barr virus are known to be closely related to AIH type 1 [30]. We searched related literature with the MeSH term “Hepatitis, Autoimmune” and “Hepatitis A” in the MEDLINE database. Although the condition of AIH along with hepatitis A is rare, it has been reported globally; 14 cases have been reported to date, not including the current case report (Table 1). Among them, eight cases developed in young (20s) to middle-aged (50s) women. 

For all the reported cases of AIH along with hepatitis A, patients tested positive for ANA or SMA while negative for anti-LKM and anti-LC1, which are the characteristics of type 2 AIH. It took varying amount of time for the diagnosis of AIH after the onset of hepatitis A, ranging from 2 months to 19 months. Though immunosuppressive treatment such as steroid and azathioprine was necessary in 13 cases, 1 case of an asymptomatic patient was followed-up without treatment. AIH relapsed in 3 cases, and a small amount of immunosuppressant was maintained, even after the resolution of AIH in most cases.

Consistently with the extant AIH cases of middle-aged women, the current case report focuses on the 55-year-old patient who developed AIH after hepatitis A. The patient tested positive for ANA, which indicates type 1 AIH. Genetic and immunological sensitivities were not evaluated because neither HLA type nor ASGPR was examined. Meanwhile, the interval between the diagnosis of hepatitis A and AIH was about 1 month, which was shorter than those of the other reported cases. Additionally, in contrast to other reported cases, the patient of the current report did not need immunosuppressants, and conservative treatments were maintained due to a lack of severe symptoms and mildly elevated levels in liver enzymes.

After the comprehensive review of the extant AIH case reports, including the current one, we recommend not to rule out the possibility of AIH after the onset of hepatitis A and close monitoring over time, especially in the following conditions: (i) persistent symptoms such as mild fever and fatigue or continuous aminotransferase elevation after the recovery period, (ii) relapse of symptoms such as jaundice, fatigue, hyperbilirubinemia, and aminotransferase elevation, (iii) being a young or middle-aged woman with a history of hepatitis A, and (iv) hypergammaglobulinemia or tested positive for ANA or SMA but did not fulfill the AIH scoring system at the time of the hepatitis A diagnosis. 

## Figures and Tables

**Table 1:** Characteristics of acute hepatitis A-induced autoimmune hepatitis based on prior case reports.

References	Year	Demographic and Clinical Characteristics			Course of Illness			
		Location	Sex	Age (yr)	Symptom	Antibodies	Treatment	Outcome
Vento et al. [28]	1991	Italy	M	18	None	Anti- ASGPR/ANA Actin-antibodies	Methylprednisolone Azathioprine	Resolved
Vento et al. [28]	1991	Italy	F	13	None	Anti- ASGPR/ANA Actin-antibodies	Methylprednisolone Azathioprine	Resolved
Rahaman et al. [31]	1994	USA	F	55	Fatigue Jaundice	ANA	Prednisone	Relapsing
Huppertz et al. [32]	1995	Germany	F	7	Fatigue Jaundice	SMA Anti-ASGPR	Prednisolone Azathioprine	Resolved
Hilzenrat et al. [12]	1999	Israel	F	55	Fatigue Jaundice	SMA	Prednisone	Resolved
Wozniakowska-Gesicka et al. [33]	2001	Poland	F	13	?	ANA SMA	Prednisone	Resolved
Munoz Bertran et al. [34]	2002	Spain	M	27	Jaundice	ANA SMA	Prednisone Later azathioprine	Response
Skoog et al. [35]	2002	USA	F	24	Nausea Jaundice	ANA	Prednisone Later azathioprine added	Relapsing
Tagle Arrospide et al. [36]	2003	Peru	F	61	Arthralgia	ANA SMA	No treatment	Under observation
Grünhage et al. [37]	2004	Germany	F	75	Malaise Jaundice	ANA SMA	Prednisone Azathioprine	Resolved
Tanaka et al. [5]	2005	Japan	F	57	Jaundice	ANA	Prednisolone	Resolved
Singh et al. [38]	2007	United Kingdom	F	Unknown	Nausea Dark urine	ANA	Immunosuppressants	Complete response
Tabak et al. [1]	2008	Turkey	F	21	Fever Weakness Jaundice	ANA	Prednisolone Azathioprine	Relapsing
Kim et al. [39]	2011	Republic of Korea	F	57	Fever Jaundice	ANA SMA	Prednisone Azathioprine	Resolved
Our case	2021	Republic of Korea	F	55	Weakness Jaundice	ANA	No treatment	Resolved

M = male, F = female, ANA = Antinuclear antibody, Anti-ASGPR = Anti-asialoglycoprotein receptor, SMA = Smooth muscle antibody.

## Data Availability

The datasets generated during and/or analyzed during the current study are available from the corresponding author on reasonable request.

## Abbreviations

AIH = autoimmune hepatitis, ANA = anti-nuclear antibody, HLA = human lymphocyte antigen, HAV = hepatitis A virus, HBV = hepatitis B virus, BMI = body mass index, AST = aspartate aminotransferase, ALT = aminotransferase, ALP = alkaline phosphatase, γ-GT = gamma-GTP, PT/INR = prothrombin time-international normalized ratio, Anti-HAV IgM = anti-hepatitis A virus IgM, HBsAg = hepatitis B surface antigen, Anti-HBs = anti-hepatitis B surface, Anti-HBc IgM = anti-hepatitis B core IgM, Anti-HCV = anti-hepatitis C virus, MELD = Model for End-stage Liver Disease, CT = computed tomography, SMA = smooth muscle antibody, Anti-LKM = anti-liver kidney microsomal antibody, AMA = anti-mitochondrial antibody, IgG = immunoglobulin G, MHC = major histocompatibility complex, ASGPR = asialoglycoprotein receptor, IAIHG = International Automatic Immunohepatic Group, Anti-LC1 = antibody to liver cytosol-1.

## References

[1] Tabak F., Ozdemir F., Tabak O., Erer B., Tahan V., Ozaras R. (2008). Autoimmune hepatitis induced by the prolonged hepatitis A virus infection. Ann. Hepatol..

[2] Liberal R., Longhi M.S., Mieli-Vergani G., Vergani D. (2011). Pathogenesis of autoimmune hepatitis. Best Pract. Res. Clin. Gastroenterol..

[3] Wang M., Zhang H. (2018). The pathogenesis of autoimmune hepatitis. Front. Lab. Med..

[4] Beringer A., Miossec P. (2018). IL-17 and IL-17-producing cells and liver diseases, with focus on autoimmune liver diseases. Autoimmun. Rev..

[5] Tanaka H., Tujioka H., Ueda H., Hamagami H., Kida Y., Ichinose M. (2005). Autoimmune hepatitis triggered by acute hepatitis A. World J. Gastroenterol..

[6] Giannini E.G., Testa R., Savarino V. (2005). Liver enzyme alteration: A guide for clinicians. CMAJ.

[7] Kalas M.A., Chavez L., Leon M., Taweesedt P.T., Surani S. (2021). Abnormal liver enzymes: A review for clinicians. World J. Hepatol..

[8] Manns M.P., Czaja A.J., Gorham J.D., Krawitt E.L., Mieli-Vergani G., Vergani D., Vierling J.M. (2010). Diagnosis and management of autoimmune hepatitis. Hepatology.

[9] Czaja A.J. (2001). Understanding the pathogenesis of autoimmune hepatitis. Am. J. Gastroenterol..

[10] Czaja A.J., Strettell M.D., Thomson L.J., Santrach P.J., Moore S.B., Donaldson P.T., Williams R. (1997). Associations between alleles of the major histocompatibility complex and type 1 autoimmune hepatitis. Hepatology.

[11] Strettell M.D., Donaldson P.T., Thomson L.J., Santrach P.J., Moore S.B., Czaja A.J., Williams R. (1997). Allelic basis for HLA-encoded susceptibility to type 1 autoimmune hepatitis. Gastroenterology.

[12] Hilzenrat N., Zilberman D., Klein T., Zur B., Sikuler E. (1999). Autoimmune hepatitis in a genetically susceptible patient: Is it triggered by acute viral hepatitis A?. Dig. Dis. Sci..

[13] Vento S., Garofano T., Di Perri G., Dolci L., Concia E., Bassetti D. (1991). Identification of hepatitis A virus as a trigger for autoimmune chronic hepatitis type 1 in susceptible individuals. Lancet.

[14] Alvarez F., Berg P.A., Bianchi F.B., Bianchi L., Burroughs A.K., Cancado E.L., Chapman R.W., Cooksley W.G., Czaja A.J., Desmet V.J. (1999). International Autoimmune Hepatitis Group Report: Review of criteria for diagnosis of autoimmune hepatitis. J. Hepatol..

[15] Krawitt E.L. (2006). Autoimmune hepatitis. N. Engl. J. Med..

[16] Zhang W.C., Zhao F.R., Chen J., Chen W.X. (2014). Meta-analysis: Diagnostic accuracy of antinuclear antibodies, smooth muscle antibodies and antibodies to a soluble liver antigen/liver pancreas in autoimmune hepatitis. PLoS ONE.

[17] Czaja A.J., Freese D.K. (2002). American Association for the Study of Liver Disease. Diagnosis and treatment of autoimmune hepatitis. Hepatology.

[18] Gleeson D., Heneghan M.A. (2011). British Society of Gastroenterology British Society of Gastroenterology (BSG) guidelines for management of autoimmune hepatitis. Gut.

[19] Al-Hamoudi W.K. (2009). Severe autoimmune hepatitis triggered by varicella zoster infection. World J. Gastroenterol..

[20] Le Cann P., Tong M.J., Werneke J., Coursaget P. (1997). Detection of antibodies to hepatitis E virus in patients with autoimmune chronic active hepatitis and primary biliary cirrhosis. Scand. J. Gastroenterol..

[21] Castellote J., Guell E., Porta F. (2001). Autoimmune hepatitis following cytomegalovirus infection. Med. Clin..

[22] Ferri S., Muratori L., Quarneti C., Muratori P., Menichella R., Pappas G., Granito A., Ballardini G., Bianchi F.B., Lenzi M. (2009). Clinical features and effect of antiviral therapy on anti-liver/kidney microsomal antibody type 1 positive chronic hepatitis C. J. Hepatol..

[23] Hagel S., Bruns T., Herrmann A., Tannapfel A., Stallmach A. (2012). Autoimmune hepatitis in an HIV-infected patient: An intriguing association. Int. J. STD AIDS.

[24] Kamisako T., Tsubaki K., Adachi Y. (1997). Autoimmune hepatitis after cytomegalovirus infection in a bone marrow-transplanted patient. Am. J. Gastroenterol..

[25] Laskus T., Slusarczyk J. (1989). Autoimmune chronic active hepatitis developing after acute type B hepatitis. Dig. Dis. Sci..

[26] Obermayer-Straub P., Manns M.P. (2001). Hepatitis C and D, retroviruses and autoimmune manifestations. J. Autoimmun..

[27] Toyoda-Akui M., Yokomori H., Kaneko F., Shimizu Y., Takeuchi H., Tahara K., Yoshida H., Kondo H., Motoori T., Ohbu M. (2011). Association of an overlap syndrome of autoimmune hepatitis and primary biliary cirrhosis with cytomegalovirus infection. Int. J. Gen. Med..

[28] Vento S., Guella L., Mirandola F., Cainelli F., Di Perri G., Solbiati M., Concia E., Ferraro T. (1995). Epstein-Barr virus as a trigger for autoimmune hepatitis in susceptible individuals. Lancet.

[29] Zellos A., Spoulou V., Roma-Giannikou E., Karentzou O., Dalekos G.N., Theodoridou M. (2013). Autoimmune hepatitis type-2 and Epstein-Barr virus infection in a toddler: Art of facts or an artifact?. Ann. Hepatol..

[30] Vento S., Cainelli F. (2004). Is there a role for viruses in triggering autoimmune hepatitis?. Autoimmun. Rev..

[31] Rahaman S.M., Chira P., Koff R.S. (1994). Idiopathic autoimmune chronic hepatitis triggered by hepatitis A. Am. J. Gastroenterol..

[32] Huppertz H.I., Treichel U., Gassel A.M., Jeschke R., Meyer zum Buschenfelde K.H. (1995). Autoimmune hepatitis following hepatitis A virus infection. J. Hepatol..

[33] Wozniakowska-Gesicka T., Kups J., Wisniewska-Ligier M., Wroblewska W., Sulat-Syncerek D., al-Batool K. (2001). Autoimmune hepatitis in the course of chronic HAV—Four year observation. Med. Sci. Monit..

[34] Munoz Bertran E., Rosa Salazar V., Hostalet Robles F., Correa Estan J.A., Belda Abad G., Munoz Ramirez E. (2002). Autoimmune hepatitis caused by acute hepatitis due to hepatitis A virus. Gastroenterol. Hepatol..

[35] Skoog S.M., Rivard R.E., Batts K.P., Smith C.I. (2002). Autoimmune hepatitis preceded by acute hepatitis A infection. Am. J. Gastroenterol..

[36] Tagle Arrospide M., Leon Barua R. (2003). Viral hepatitis A as a triggering agent of autoimmune hepatitis report of a case and review of literature. Rev. Gastroenterol. Peru.

[37] Grunhage F., Spengler U., Fischer H.P., Sauerbruch T. (2004). Autoimmune hepatitis—Sequel of a relapsing hepatitis A in a 75-year-old woman. Digestion.

[38] Singh G., Palaniappan S., Rotimi O., Hamlin P.J. (2007). Autoimmune hepatitis triggered by hepatitis A. Gut.

[39] Kim Y.D., Kim K.A., Rou W.S., Lee J.S., Song T.J., Bae W.K., Kim N.-H. (2011). A case of autoimmune hepatitis following acute hepatitis A. Korean J. Gastroenterol..

